# Evaluation of postoperative refractive error correction after cataract surgery

**DOI:** 10.1371/journal.pone.0252787

**Published:** 2021-06-17

**Authors:** Ellen Konadu Antwi-Adjei, Emmanuel Owusu, Emmanuel Kobia-Acquah, Emmanuella Esi Dadzie, Emmanuel Anarfi, Seth Wanye

**Affiliations:** 1 Department of Optometry and Visual Science, Kwame Nkrumah University of Science and Technology, Kumasi, Ghana; 2 Friends Eye Center, Kumasi, Ghana; University of Toronto, CANADA

## Abstract

Suboptimal cataract surgery outcomes remain a challenge in most developing countries. In Ghana, about 2 million people have been reported to be blind due to cataract with about 20% new cases being recorded yearly. The aim of this study was to evaluate postoperative correction of refractive errors after cataract surgery in a selected eye hospital in Ashanti Region, Ghana. This was a retrospective study where medical records of patients (aged 40–100) who reported to an eye hospital in Ghana from 2013–2018 were reviewed. Included in the study were patients aged ≥40 years and patients with complete records. Data on patient demographics, type of surgery, intra-ocular lens (PCIOL) power, availability of biometry, postoperative refraction outcomes, pre- and postoperative visual acuity were analyzed. Data of two hundred and thirteen eyes of 190 patients who met the inclusion criteria were analyzed. Descriptive analysis and Chi-square test were carried out to determine the mean, median, standard deviation and relevant associations. The mean ± *SD* age was 67.21±12.2 years (51.2% were females). Small Incision Cataract Surgery (99.5%) with 100% IOL implants was the main cataract surgery procedure in this study. Pre-operative biometry was performed for 38.9% of all patients on their first eye surgery and 41.5% for second eye surgeries. About 71% eyes in this study were blind (presenting VA<3/60) before surgery; 40.4% had post-operative VA <3/60. Pre-existing ocular comorbidities discovered post- surgery, attributed to suboptimal visual outcomes. More than half (55.3%) of patients did not undergo postoperative refraction due to loss to follow-up. Year of surgery (p = .017), follow up visits< 2months (p < .0001) and discovered comorbidity post-surgery (p = .035) were the factors significantly associated with postoperative refraction. Myopia and compound myopic astigmatism were the dominant refractive error outcomes. The timing of post-operative refraction had a significant effect on postoperative refraction done. These findings indicate a clinically meaningful significance between completion of postoperative care and postoperative refraction done. Consequently, with settings in most developing countries, where less biometry is done, it is appropriate that post-operative refractive services are encouraged and done earlier to enhance the patients’ expectations while increasing cataract surgery patronage.

## Introduction

More than 90% of the global burden of blindness from cataract can be found in developing countries [1, 2]. In Ghana, cataract is responsible for approximately 55% of blindness [presenting visual acuity (VA) < 3/60] [3]. Although surgical extraction remains the best intervention for blinding cataract, residual refractive errors are common after surgery. Thus, refractive error correction after cataract surgery is often necessary as high magnitudes of residual refractive errors are a major obstacle to successful surgical outcomes. Consequently, there is a need for more accurate intra-ocular lens (IOL) power estimation in order to minimise the resulting residual refractive errors and maximize surgical benefits. In addition to increasing cataract surgical rates, high quality of cataract surgical outcomes contributes to achieving targets related to the Sustainable Development Goals of universal health care [4].

Advancement in cataract surgery has been synonymous with the development of algorithms for better estimation of the power of intraocular lens (IOL) that should be implanted in the eye using biometry [5]. In addition to improved IOL implantation, surgical procedures are now more accurate. However, even in the hands of the most qualified and meticulous surgeons, refractive errors can occur due to numerous factors [6]. Refractive surprises are commonly found in situations where the gold standard for calculating the intraocular lens power has not been implemented [7], and may manifest as hyperopia, myopia or astigmatism. Refractive error correction after cataract surgery may vary from noninvasive options like spectacle correction to the more complex surgical correction options such as laser vision correction and IOL exchange [7].

Reports on the use of average-power IOLs (without biometry) in cataract programs in the developing world have reported good visual outcomes with easier management of inventory hence not purchasing and maintaining an ultrasound unit becomes meritorious [8]. However, population—and hospital—based studies have shown that the outcomes of cataract surgery in many low and middle–income (LMICs) countries are frequently sub-optimal, often below the recommended standards set by the World Health Organization (WHO) [9–11]. Those with poor vision, following cataract surgery are less likely to have an improved quality of life [10, 12, 13].

Data on cataract monitoring from Ghana are similarly scarce, particularly regarding visual outcomes and postoperative refraction after cataract surgery [13, 14]. The results of one study in Ghana showed that although 90% of people who had undergone cataract surgery had residual refractive errors, they did not have any spectacle correction [13]. We present a study on the surgical approaches employed and postoperative refractive error correction after cataract surgery in a developing country. This study intended to evaluate cataract surgical outcomes by investigating the residual refractive errors and their correction among a group of patients who have undergone cataract surgery in an eye hospital in Ghana from 2013 to 2018. Specifically, the study investigated the proportion of patients who underwent the different cataract surgical approaches–Extra Capsular Cataract Extraction (ECCE), Manual Small Incision Cataract Surgery (MSICS) and Phacoemulsification. Also, the proportion of patients who underwent post-surgical refractive correction, and at what period after the surgery were identified. Finally, the features of residual refractive errors and their correction at final discharge (8–10 weeks post-surgically) were investigated.

Although other studies [11, 13, 15, 16] have reported on the visual outcomes after cataract surgery, none of these have focused on the postoperative refraction services and optical correction outcomes. The outcomes of the present study may provide data to support quality improvement initiatives to maximize surgical benefits for enhancing socioeconomic and quality of life benefits.

## Materials and methods

Data were collected retrospectively from hospital folders of patients who had undergone cataract extraction from the same cataract surgeon at a private eye hospital in the Kumasi metropolis. Data extracted from the folders were sex, age, cataract surgery technique, IOL power selection methods and postoperative refraction outcomes (S1 Table). Three events were considered in this study. The first event was the type of cataract surgery employed (IOL power selection methods; biometry and implantation). The second event was the duration of follow-up period and postoperative refractive service, which was confirmed through the presence of objective and subjective refraction (as stated in the patient folders). The last event was to characterize the outcomes after postoperative refraction (if done); which was confirmed through the presence of the BCVA and the refractive error as stated in the patient folders. The clinical records of all such patients that underwent cataract surgery between January 2013 and December 2018 inclusive were reviewed. The patients who had complications (e.g. uveitic cataract) and incomplete records were excluded (S1 Fig).

Preoperative data was collected on best corrected visual acuity (BCVA), refraction, surgery eye/order, pre-existing ocular comorbidity expected to reduce postoperative acuity to 6/12 or worse and biometry. The parameters indicated was to investigate if intraocular lens (IOL) implantation was done or not, whether the axial length and keratometric readings of these eyes were taken to calculate the precise power of IOL to be implanted. Biometry was obtained through the contact method (Sonomed INC. 5500 Ultrasound A-scan machine), K-readings were measured using a Bausch & Lomb Keratometer (Bausch & Lomb, USA.), and the IOL power was calculated using the SRK/T formula. Average-power IOL was chosen for patients that did not have biometry done. The IOL power was predicted using patients’ age, gender and in certain cases the pre-operative refraction status. Thus the ophthalmologist considers the presenting vision and other factors such as existing comorbidities to select the IOL. This is done under the sole prerogative of the ophthalmologist and is mostly based on long years of experience.

To allow comparison with other studies, a visual acuity threshold of 6/12 or better was chosen to define the surgical outcome. WHO also has recommended and set targets aimed at achieving good uncorrected visual acuity (UCVA) in at least 80% of surgeries and poor in less than 5% and corrected visual acuity of good in 90% of surgeries and poor in less than 5% by 2 months after surgery [17, 18].

All statistical analyses were performed using Statistical Package for the Social Sciences (SPSS) software version 23.0 (IBM Corporation, Armonk, NY, USA). The level of statistical significance was set at p≤ 0.05 with 95% confidence.

Ethical approval was sought from the Committee on Human Research, Publication & Ethics (CHRPE) of Kwame Nkrumah University of Science and Technology, Kumasi, Ghana. All study protocols conformed to the Tenets of the Declaration of Helsinki.

## Results

Data on 213 eyes from 190 patients, 55.9% of whom were females met the eligibility criteria and were included in this study. The mean age of the participants was 67.21±12.2 years (mean ± *S.D*), with a range of 40–100 years and mostly within the 70-79-year age group. Table 1 shows the details of age, sex and occupational distribution of the participants.

**Table 1:** Socio-demographic characteristics of the patients studied.

		Sex of Patient		
		Male	Female	Total
		N = 104	N = 109	N = 213
		n (%)	n (%)	n (%)
	**Age (years)**			
	40–49	14(13.5)	3(2.8)	17(8.0)
	50–59	25(24.0)	19(17.4)	44(20.7)
	60–69	15(14.4)	28(26.7)	43(24.7)
	70–79	36(34.6)	38(34.9)	74(34.7)
	80–89	11(10.6)	17(15.6)	28(13.1)
	90–100	3(2.9)	4(3.7)	7(3.3)
	**Occupation**			
	Trading	5(4.8)	15(13.8)	20(9.4)
	Farming	15(14.4)	8(7.3)	23(10.8)
	Driving	10(9.6)	0(0.0)	10(4.7)
	Teaching	6(5.8)	4(3.7)	10(4.7)
	Clerk	9(8.7)	0(0.0)	9(4.2)
	Others	3(2.9)	2(1.8)	5(2.4)
	Unemployed	4(3.8)	13(11.9)	17(7.9)
	Not recorded	52(50.0)	67(61.5)	119(55.9)

### Cataract surgical technique & IOL implantation

**Fig 1:**
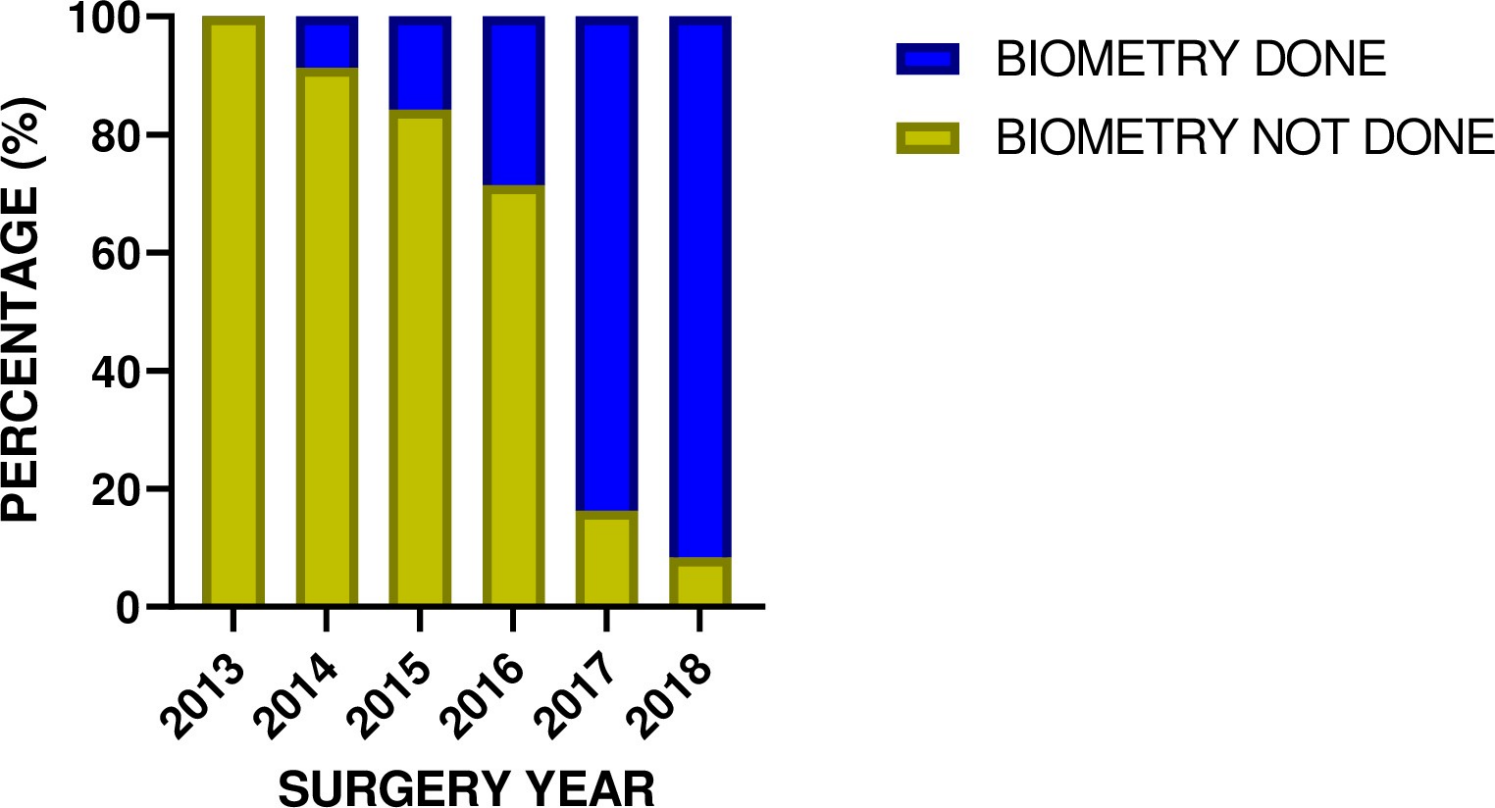
Distribution of biometry done over the years under study.

All operated eyes were implanted with IOL. Also, all but one underwent manual small incision cataract surgery (MSICS) by the same surgeon throughout the study period. That patient underwent extracapsular cataract extraction. However, not every patient underwent biometry prior to undergoing surgery: as the years advanced, the number of biometry performed increased (Fig 1). There was a statistically significant association between the year surgery was performed and whether biometry was performed or not (χ^2^ = 1110.510, p<0.0001) (Fig 1). In all, biometry was performed for 38.9% of the eyes studied (Table 2).

**Table 2:** Cataract surgical technique used over the years studied.

		Biometry		P-value
		Done	Not Done	
		N = 83	N = 130	
		n (%)	n (%)	
Surgery Order	First Eye	61(73.5)	99(76.2)	0.446^a^
	Second Eye	22(26.5)	31(23.8)	
IOL Location	PC	83(100.0)	128(98.5)	0.522^b^
	AC	0(0.0)	2(1.5)	
Post operative Visual Acuity (UCVA)	NPL	0(0.0)	4(3.1)	0.248^c^
	<1/60-LP	10(12.0)	18(13.8)	
	<3/60-1/60	4(4.8)	12(9.2)	
	<6/60-3/60	14(16.9)	25(19.2)	
	<6/18-6/60	29(34.9)	43(33.1)	
	6/6-6/18	23(27.7)	26(20.0)	
	6/5	3(3.6)	2(1.5)	
Residual Refractive Errors	Myopia	8(42.1)	7(26.9)	0.445^c^
	Hyperopia	2(10.5)	2(7.7)	
	Simple myopic astigmatism	1(5.3)	3(11.5)	
	Compound myopic astigmatism	7(36.8)	9(34.6)	
	Compound hyperopic astigmatism	0(0.0)	3(11.5)	
	Mixed Astigmatism	1(5.3)	2(7.7)	

*AC = Anterior Chamber; *PC = Posterior Chamber; *ECCE = Extracapsular Cataract Extraction; *SICS = Small Incision Cataract Surgery

^a^ Chi-square test

^b^ Fisher’s exact test

^c^ Likelihood ratio chi square.

Patients who were undergoing surgery in their second eyes had a higher number of biometry done (41.5%) compared to those who had surgery in their first eyes (38.1%), although this difference was not statistically significant (χ^2^ = 0.582, p = 0.446). There was no NPL visual outcome for patients who had biometry done and majority (34.9%) had UCVA of <6/18-6/60. Majority of patients who had biometry done were most likely to have post operative refractive error service (p = 0.445) (Table 2).

### Postoperative refraction

The influence of refraction schedule, biometry, year surgery was done, surgery order and ocular comorbidities was used to evaluate the possibility of conducting postoperative refraction. Postoperative refraction was not performed for 39.9% of eyes due to incomplete postoperative care and loss to follow-up. A clinically significant relationship was found between duration since surgery and postoperative refraction (χ^2^ = 135.158, p<0.0001) with postoperative assessment done mostly 8 weeks after surgery irrespective of which eye was operated first. Ocular comorbidity appears to be a factor associated with performance of postoperative refraction (Table 3).

**Table 3:** Postoperative refraction.

Performance of Postoperative Refraction					
	Yes	No (By Completion of Post-Op visits)	Loss to Follow-Up	Total	P-value
	N = 45	N = 83	N = 85	N = 213	
	n (%)	n (%)	n (%)	n (%)	
**Duration After Surgery**					
1–3 Weeks Post-Op.	0(0.0)	0(0.0)	4(4.7)	4(1.9)	0.000^a^
4–6 Weeks Post-Op	0(0.0)	3(3.6)	20(23.3)	23(10.8)	
6–8 Weeks Post-Op	3(6.7)	1(1.2)	47(55.3)	51(23.9)	
≥ 9 Weeks Post-Op*	42(93.3)	79(95.2)	14(16.5)	135(63.4)	
**Biometry**					
Done	22(48.9)	34((41.0)	27(31.8)	83(39.0)	0.145^a^
Not Done	23(51.1)	49(59.0)	58(68.2)	130(61.0)	
**Surgery Year**					
2013	6(13.3)	7(8.2)	8(9.4)	21(9.9)	0.017^a^
2014	6(13.3)	21(25.3)	19(22.4)	46(21.6)	
2015	9(20.0)	6(7.2)	23(27.1)	38(17.8)	
2016	10(22.2)	11(13.3)	14(16.5)	35(16.4)	
2017	12(26.7)	24(28.9)	13(15.3)	49(23.0)	
2018	2(4.4)	14(16.9)	8(9.4)	24(11.3)	
**Surgery order**					
First Eye	34(75.6)	62(74.7)	64(75.3)	160(75.1)	0.035^a^
Second Eye	11(24.4)	21(25.3)	21(24.7)	53(24.9)	
**Pathological Surprises During Surgery**					
Old iritis	2(4.4)	1(1.2)	3(3.5)	6(2.8)	
Retinal disease (DM, HPT, AMD)	3(6.7)	0(0.0)	1(1.2)	4(1.9)	
Glaucoma	1(2.2)	3(3.6)	6(7.1)	10(4.7)	
Macula hole	1(2.2)	0(0.0)	0(0.0)	1(0.5)	
Other (Maculopathy, Optic atrophy. CRVO, CRAO)	6(13.3)	28(33.7)	25(29.4)	59(27.7)	
None	32(71.1)	51(61.4)	3(3.5)	133(62.4)	

*Post-Op, Postoperative

^a^, obtained by Chi-square test.

### Refractive error correction outcomes after cataract surgery

Twelve (21.1%) eyes with no pre-existing ocular pathology had BCVA at distance of 6/12 or better (Table 4). Most of the eyes had remarkable improvement in visual acuity after the postoperative refractive error correction (Table 5). The commonest refractive error was compound myopic astigmatism (35.5%) although not statistically significant but was clinically significant. This was closely followed by myopia (33.3%).

**Table 4:** The refractive error correction outcomes at post-operative follow-up.

	Corrected Visual Acuity Post-Op (BCVA)			
	≥6/12	<6/12	Total (%)	P-value^a^
	N(30)	N(15)		
	n (%)	n (%)		
**Residual Refractive Errors**				0.409
Myopia	10(33.3)	5(33.3)	15(33.3)	
Hyperopia	1(3.3)	3(20.0)	4(8.9)	
Simple myopic astigmatism	2(6.7)	2(13.3)	4(8.9)	
Compound myopic astigmatism	12(40.0)	4(26.7)	16(35.5)	
Compound hyperopic astigmatism	2(6,7)	1(6.7)	3(6.7)	
Mixed astigmatism	3(10.0)	0(0.0)	3(6.7)	
**Post-operative Visual Outcome (UCVA)**				0.009
<3/60-LP (Poor outcome)	2(6.6)	3(20.0)	5(11.1)	
<6/60-3/60 (Poor outcome)	3(10.0)	7(46.7)	10(22.2)	
<6/18-6/60 (Borderline outcome) *	10(33.3)	5(33.3)	15(33.3)	
6/5-6/18 (Good outcome) *	15(50.0)	0(0.0)	15(33.3)	
**Pre-Operative Biometry**				0.393
Yes	14(46.7)	5(33.3)	19(42.2)	
No	16(53.3)	10(66.7)	26(57.7)	

*WHO guidelines on visual outcome (VA) post operatively: good outcome: 6/6-6/18, borderline: < 6/18-6/60, poor: <6/60

^a^, p-value obtained by Chi-square.

**Table 5:** Comorbidities and postoperative refraction.

Performance of Postoperative Refraction					
	Yes	No (By Completion of Post-Op visits)	Loss to Follow-Up	Total	P-value
	N = 45	N = 83	N = 85	N = 213	
	n (%)	n (%)	n (%)	n (%)	
**Pre-existing ocular comorbidity**					
None	15(33.3)	20(24.1)	22(25.9)	57(26.8)	0.412^a^
Cornea scar	0(0.0)	1(1.2)	1(1.2)	2(0.9)	
Old iritis	0(0.0)	0(0.0)	0(0.0)	0(0.0)	
Retinal disease (DM,HPT,AMD)	4(8.9)	2(2.4)	2(2.4)	8(3.8)	
Glaucoma	4(8.8)	3(3.6)	8(9.4)	15(7.0)	
Other (Kerathopathy, Papilledema, Toxoplasmosis, Chorioretinitis etc)	1(2.2)	6(7.2)	6(7.1)	13(6.1)	
Poor view (undiagnosed)	20(44.5)	51(41.4)	45(52.8)	116(54.5)	
Glaucoma with Retinal Disease	1(2.2)	0(0.0)	1(1.2)	2(0.9)	
**Intra-Operative Complication**					
None	43(95.6)	79(95.2)	83(97.6)	205(96.2)	0.896^a^
Capsular rupture without vitreous loss	0(0.0)	1(1.2)	0(0.0)	1(0.5)	
Vitreous loss	1(2.2)	1(1.2)	1(1.2)	3(1.4)	
Others (Retained lens matter, keratopathy)	1(2.2)	2(2.4)	1(1.2)	4(1.8)	
**Early complications**					
None	26(57.8)	38(45.8)	38(44.7)	102(47.9)	0.320^a^
Cornea haziness	9(20.0)	17(20.5)	18(21.2)	44(20.7)	
Descemets folds	1(2.2)	5(6.0)	3(3.5)	9(4.2)	
Hyphema	1(2.2)	5(6.0)	9(10.6)	15(7.0)	
Hypopyon	0(0.0)	3(3.6)	2(2.4)	5(2.3)	
Cornea haziness and descement folds	0(0.0)	3(3.6)	7(8.2)	10(4.7)	
Inflammatory cells	3(6.7)	3(3.6)	2(2.4)	8(3.8)	
Dilated pupils	1(2.2)	3(3.6)	4(4.7)	8(3.8)	
Distorted pupil	3(6.7)	1(1.2)	1(1.2)	5(2.3)	
Retained cortical matter	0(0.0)	2(2.4)	0(0.0)	2(0.9)	
Poor wound healing	1(2.2)	3(3.6)	1(1.2)	5(2.3)	
**Late complications**					
None	40(88.9)	65(78.3)	67(78.8)	172(80.8)	0.591^a^
Posterior capsular opacification (PCO)	0(0.0)	0(0.0)	3(3.5)	3(1.4)	
Increased IOP	0(0.0)	2(2.4)	1(1.2)	3(1.4)	
Endolphthalmitis	0(0.0)	2(2.4)	1(1.2)	3(1.4)	
Ptosis	0(0.0)	1(1.2)	0(0.0)	1(0.5)	
Iridocyclitis	1(2.2)	4(4.8)	1(1.2)	6(2.8)	
Lens displacement	0(0.0)	1(1.2)	2(2.3)	3(1.4)	
Kerathopathy	1(2.2)	2(2.4)	5(5.9)	8(3.8)	
Iris pigment deposits on IOL	1(2.2)	1(1.2)	3(3.5)	5(2.3)	
Inflammatory cells in vitreous	0(0.0)	2(2.4)	1(1.2)	3(1.4)	
Retinal hemorrhages	0(0.0)	1(1.2)	1(1.2)	2(0.9)	
Cystic macula edema	1(2.2)	0(0.0)	0(0.0)	1(0.5)	
Iridocyclitis and PCO	0(0.0)	1(1.2)	0(0.0)	1(0.5)	
Decreased IOP	1(2.2)	1(1.2)	0(0.0)	2(0.9)	

*Post-Op, Postoperative

^a^, obtained by Chi-square test.

## Discussion

The eye facility used in this study mainly performed MSICS throughout the study period. Apart from one eye which underwent ECCE, all other eyes were operated on using MSICS. These results are similar to findings from other studies that showed that MSICS with IOL implantation is increasingly being adopted in developing countries [1, 9, 13]. Compared to SICS, MSICS and phacoemulsification, the ECCE procedure is almost obsolete as it is most likely to provide the worst visual outcome after surgery among the other surgical techniques. SICS has been demonstrated to give better uncorrected visual acuity [15] in a greater proportion of patients, is less expensive and almost as effective as phacoemulsification, the gold standard.

Less than half of the surgeries performed within the study period had prior biometry. Biometry was performed for nearly 40% of eyes studied, with a higher tendency for biometry in the more recent years. Although this was higher than the percentage of biometry done for patients who underwent MSICS in previous studies [9, 19], it was lower when compared to the studies reported by Briesen [11] in Kenya and Farmer [1] in Bali; Indonesia–where biometry was done in all (100%) eyes-. The lack of preoperative biometry observed in this study from previous years (from 2013 to 2016) can be attributed to unavailability of equipment. The increasing performance of biometry in more recent years of the study period (2017 to 2018) may be explained by emerging trends, advancement in technology with the availability of equipment in later years, and the continuous desire for quality improvement in cataract surgical outcomes. The results further showed a strong association between biometry and visual outcome after refraction. This is presumably because biometry helps predict the accurate power of IOL to be implanted [10]. This confirms earlier research findings that showed that biometry is a good predictor for better visual outcomes. The finding in this study agrees with the suggestions made by other studies, Ilechie et al. [13] and Briesen et al. [11], on the need for accurate IOL power estimation with spectacle correction to enhance better visual outcomes so as to increase the overall benefits of cataract surgery.

The visual outcomes of cataract surgery were mostly good, with a significant reduction in the proportion of blindness after surgery. Based on the WHO’s definition of blindness as presenting visual acuity of 3/60 or worse, 71.4% of eyes were blind prior to surgery with approximately 22% remaining blind after surgery. While the proportion of blind eyes after surgery does not appear impressive, there is still an encouraging reduction in blindness. The remaining blindness is largely due to the advanced state of the cataracts prior to surgery which limited the visual potential of those eyes. Very matured cataract is more likely to be associated with complications from the cataract such a secondary glaucomas (pharcomorphic, pseudoexfoliation, etc) [19]. Besides these, advanced cataract makes it more challenging to identify comorbidities which can worsen visual prognosis. In this study, 41.2% of eyes presented with pathological surprises which were only noticed during or after cataract surgery. Advanced presentation of cataract may be due to several factors, mainly poor access to surgical opportunities or reluctance to uptake surgical interventions. It is not unusual for patients with matured cataract to decline surgery in hopes of getting medication or glasses to manage cataract. This is consistent with previous findings about people’s reluctance to undergo surgery until a great vision loss due to matured cataract [9, 10, 20]. Matured cataract comes with biometry contraindication and undetected ocular comorbidities and as a result contribute to poor case selection. In a previous study, Lindfield et al. [10] suggested early surgical intervention to help address the challenges in improving the outcome of cataract surgery in Lower Middle Income Countries. Similarly, the most common reason for poor outcomes in the Lindfield et al [10] study and Yorston [21] was the presence of pre-existing ocular pathology in the eyes operated on. Nevertheless, less ocular comorbidities were discovered post-operatively in this study (41.2%) compared to findings (58%) from the study by Lindfield’s et al. [10].

The results of this study show that by the 8^th^ week after surgery, over 55% of patients were lost to follow-up, even though the usual duration of post-operative follow-up is 6–8 weeks. Thus, those patients did not go through postoperative refraction. This is presumably because of the long period in waiting for operative inflammation to subside and for wound healing to take place before postoperative refraction is carried out. Even though the reported number of patients in this study who had postoperative refraction was less than those reported by Briesen [11], that study also reported a decline in the number of patients that turn up for follow-up visits. Also, Ilechie et al. [13] and Beyiah [20] reported 9.6% of eyes had refraction done at 4-6weeks postoperative visit but only 6% of eyes were refracted at 6 weeks postoperative visit respectively. Consequently, results of this study are consistent with the idea that although subjective refraction at 6–10 weeks after surgery is the gold standard in evaluation of refractive outcomes, very few patients return after such a time for follow-up in rural areas in Africa [11]. Perhaps, the improvement in visual acuity that patients experience is enough for them, considering that the pre-surgical visual acuities tend to be very poor. This trend in loss to follow-up may be an indication for a review of the duration of follow-up, and at what point of follow-up post-operative refraction needs to be performed. Also, the highest percentage of eyes (42.2%) that had biometry were among those who had no postoperative refraction done. The better visual acuity with greater satisfactory outcome for the patients possibly led them being lost to follow up.

The post-operative BCVA appeared to be related to the performance of pre-surgical biometry. Of the 45 eyes which received postoperative refraction, approximately 42.2% had biometry. Among the eyes which were post-operatively refracted, two-thirds (66.7%) had best corrected visual acuity (BCVA) of 6/12 or better. Comparatively, the proportion without biometry who obtained BCVA of 6/12 or better was 53.3%. The BCVA results from this study was lower than that reported from previous studies [11, 20]. This appears to be partly due to the differences in the number of eyes that had biometry before surgery and also the percentage with ocular comorbidities in the various studies. Preoperative assessment of the eyes revealed 54.5% (116 out of 213) had undetermined pre-existing co-pathology due to poor view. Out of the 17.2% eyes (20 out of 116) which had postoperative refraction done, best-corrected distance visual acuity worse than 6/12 was reported in 50% (10 out of 20) eyes. This could explain why refraction was not done even after completion of follow-up visits. Our research indicates that majority of subjects had postoperative compound myopic astigmatism 35.5% closely followed by myopia 33.3% which is consistent with reports in other studies [11, 21]. Our study agrees with the role of biometry in reducing undesired postoperative hyperopia [11]. BCVA of 6/12 or better was reported in 46.7% of eyes that had biometry. This was lower than the 92.7% of 124 refracted eyes reported by Ilechie et al. [13], although the percentage of eyes that had biometry was not stated in their study.

In this study, vitreous loss as an intraoperative complication (1.4%) was within WHO recommendations of not more than 5% [18]. The commonest early complication was of cornea origin (39.3%) and was similar to that expressed in previous studies [13, 22]. Posterior capsular opacity (PCO) was found in only 1.8% of eyes. This late complication was higher than that reported by Ilechie et al. [13] and 1.3% in Guirou et al. [9] studies. However, it was less than the 6.3% reported by Isawuni et al. [16] and 3.3% in Beyiah [20] studies. This may be attributed to the differences in sample size for each study and the percentage of people completing the postoperative care.

The age range of ≥ 40years, where most cataract patients fall therein, makes senile cataract the most common type of cataract in developing countries, with more residual refractive errors as well. The majority of the affected people make up the working class of the country, hence better refractive outcomes postoperatively, will mean better sight and vision, enhancement in quality of work done and an overall increase in productivity of the individual and economy of the country. This will go a long way in helping attain sustainable development goal of eradicating poverty and enhancing livelihood of the citizens as a country.

### Limitations

A major limitation of our study was the fact that refractive outcomes were not determined prior to the surgery. Thus, it is not possible to determine whether the post-surgical refractive results were intended or not. Sometimes, cataract surgeons may decide to leave the patient myopic for occupational, lifestyle and quality of life reasons. It is possible to decide to leave a patient who is a high myope with a smaller magnitude of myopia. That tends to cause less disruption in the patient’s refractive demands and visual lifestyles. This notwithstanding, it is unlikely that patients will be left astigmatic or hyperopic as these refractive errors place accommodative demands on such patients. Cataract surgery eliminates the ability to accommodate so astigmatism and hyperopia would be contraindicated in cataract surgery. Therefore, despite the lack of explicit pre-surgical refractive error intentions, it is probable that all the astigmatisms identified were unintended. Further, the absence of pre-surgical refractive errors preclude the comparison of pre-surgical and post-surgical refractive errors.

## Conclusion

We found greater percentages of biometry not done and postoperative uncorrected refractive errors for the cataract surgeries studied. The timing of post-operative refraction had a significant effect on uncorrected refractive errors, where compound myopic astigmatism was the dominant refractive error outcome. Postoperative refractive services should be done earlier and promptly to increase patient corporation and positive surgical outcomes. The high number of pre-existing ocular comorbidities may have contributed to the suboptimal surgical outcomes and are likely to affect postoperative refractive error correction. In order to improve both quality and quantity of cataract surgeries and its outcomes, as we try to eliminate backlogs, the choice of informing patients about the need to complete postoperative care and carrying out postoperative refraction is very important as that can influence visual outcomes as well as encouraging cataract surgery patronage. This would help even better in the needed priority-setting, health care planning, and further investment in eye-health services. Further studies into the quality of life associated with cataract surgery on the larger population is recommended.

## Supporting information

S1 Fig Strobe flow chart. (PDF)

S1 Table Data collection form. (PDF)

S1 File Dataset. (SAV)
