# Supplementary figures and images for: Evaluation of postoperative refractive error correction after cataract surgery
Source: PLoS One. 2021 Jun 17;16(6):e0252787. doi: 10.1371/journal.pone.0252787 (PMC8211256; doi:10.1371/journal.pone.0252787)

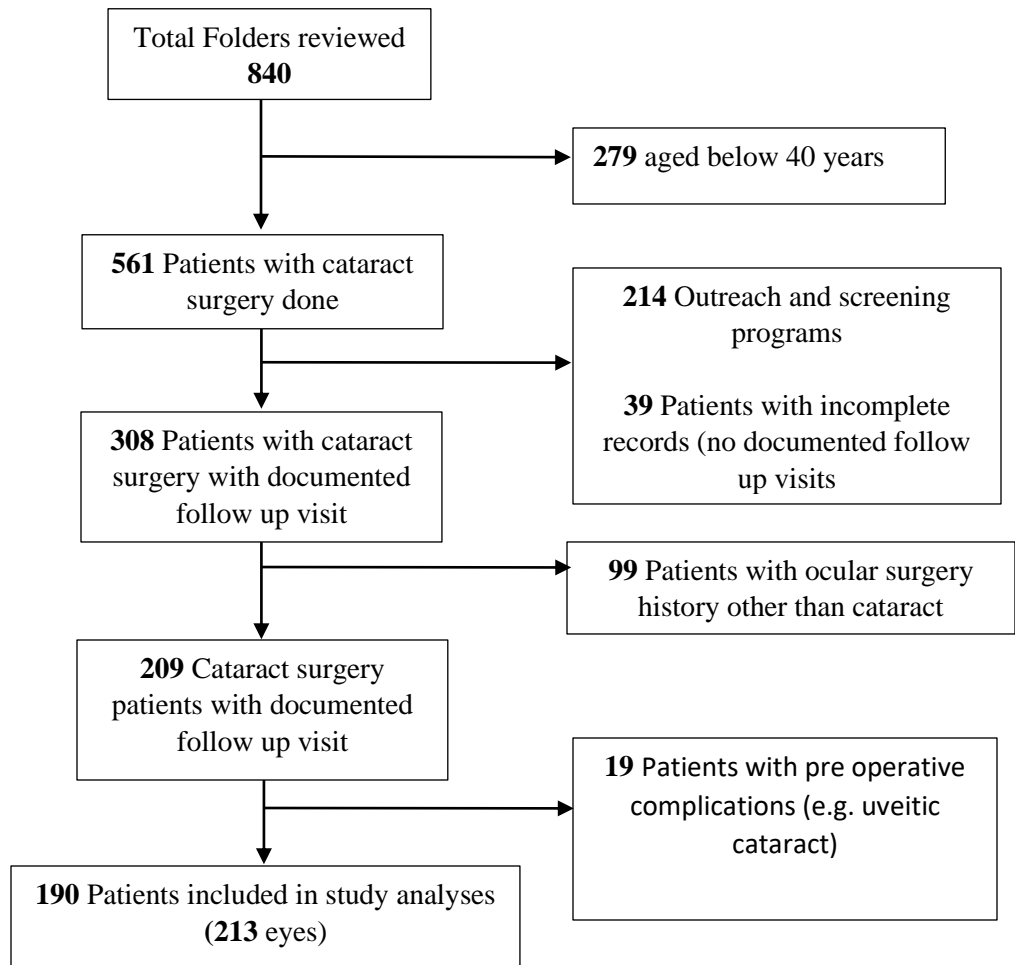

**S1 Fig. Flowchart summarizing patient eligibility and number included in study analyses**

Supplement: S1 Fig — (PDF) [file pone.0252787.s001.pdf]
