# Supplementary material for: Evaluation of postoperative refractive error correction after cataract surgery
Source: PLoS One. 2021 Jun 17;16(6):e0252787. doi: 10.1371/journal.pone.0252787 (PMC8211256; doi:10.1371/journal.pone.0252787)
Supplement: S1 Table — (PDF) [file pone.0252787.s002.pdf]

DATA COLLECTION FORM (*This information will be obtained from patient's hospital records*)

STUDY: EVALUATION OF POST –OPERATIVE REFRACTIVE ERROR AFTER CATARACT SURGERY.

1. Patient ID_____ 2.Age_____

3.Gender Male ☐Female ☐ 4. Referred ☐

	**OD**	**OS**
5. Visual Acuity: Presenting VA	_______	______
6. Best /PH VA	_______	_______
7. Surgery eye	1ST ☐	2ND ☐

8.	Lens examination:	1. Clear lens ☐<br>2. Opacity☐<br>3. Inoperable cataract☐<br>4. Pseudophakia☐<br>5. Aphakia☐<br>6. Cannot examine☐<br>7. Operable cataract☐		
9.	Other ocular pathology (Comorbidity) in the eye to be operated, likely to affect outcome:	1. None ☐<br>2. Corneal scar☐<br>3. Old irits ☐<br>4. Retinal disease (DM, HPT, AMD)☐<br>5. Glaucoma☐<br>6. Other ☐		

10. Refraction pre-operative: Eye to be operated **OD** ☐ **OS** ☐

11. Refraction sph____ cyl ____ axis___

13. Targeted post-op spherical equivalent sph _____

12. Biometry K1_______ K2 _______ NOT DONE☐

14. Axial length _______

15. SURGERY Type of surgery:	1. ECCE☐<br>2. ICCE☐<br>3. SICS☐<br>4. PHACO ☐		
16. IOL:	1. PC IOL ☐<br>2. AC IOL ☐<br>3. No IOL ☐		

17. Operative complications in operated eye	1. None ☐<br>2. Wound leak☐<br>3. Capsule rupture without vitreous loss☐<br>4. Keratopathy☐<br>5. Vitreous loss ☐<br>6. Endophthalmitis ☐<br>7. Zonular dehisce ☐<br>8. Retained lens matter ☐<br>9. Others ☐		
18. Suture	1. No suture ☐<br>2. Continuous suture☐<br>3. Interrupted suture ☐		

19. IOL power ______

20. VISUAL ACUITY OF OPERATED EYE POST-OP

20a. Follow up visits			20b. Presenting VA	
**AT DISCHARGE**	**Done**	**Not Done**	OD	OS
1-3WKS PO	☐	☐	_______	_______
4-6WKS PO	☐	☐	_______	_______
6- 8WKS PO	☐	☐	_______	_______
9 ≥ WKS PO	☐	☐	_______	_______

20c. **POST-OP REFRACTION** DONE ☐ NOT DONE ☐

20d. **Distance** **Near**

sph ____ cyl _____ axis ______ ________

sph ____ cyl _____ axis _____ ________

sph _____ cyl _____ axis _____ ________

21. **POST-OP CARE** Discovered co-morbidity:	1. Old irits<br>2. Retinal disease (DM, HPT, AMD) ☐<br>3. Glaucoma ☐<br>4. Macular hole ☐<br>5. Other ☐<br>6. None ☐		

22. Early complications ________________________________

23. Late complications________________________________

24. Self-reported visual function ________________________

25. Refractive error ________________________________

26. Surgery year ___________________________________

27. Occupation ____________________________________
